# H.U.B city steps: methods and early findings from a community-based participatory research trial to reduce blood pressure among african americans

**DOI:** 10.1186/1479-5868-8-59

**Published:** 2011-06-10

**Authors:** Jamie M Zoellner, Carol C Connell, Michael B Madson, Bo Wang, Vickie Blakely Reed, Elaine Fontenot Molaison, Kathleen Yadrick

**Affiliations:** 1 Department of Human Nutrition, Foods and Exercise, Virginia Tech, 1981 Kraft Drive (0913), Blacksburg, VA 24061, USA; 2 Department of Nutrition and Food Systems, The University of Southern Mississippi, 118 College Drive Box #5172, Hattiesburg, MS 39406-0001, USA; 3 Department of Psychology, The University of Southern Mississippi, 118 College Drive Box #5025, Hattiesburg, MS 39406-0001, USA; 4 Department of Community Health Sciences, The University of Southern Mississippi, 118 College Drive Box #5122, Hattiesburg, MS 39406-0001, USA

## Abstract

**Background:**

Community-based participatory research (CBPR) has been recognized as an important approach to develop and execute health interventions among marginalized populations, and a key strategy to translate research into practice to help reduce health disparities. Despite growing interest in the CBPR approach, CBPR initiatives rarely use experimental or other rigorous research designs to evaluate health outcomes. This behavioral study describes the conceptual frameworks, methods, and early findings related to the reach, adoption, implementation, and effectiveness on primary blood pressure outcomes.

**Methods:**

The CBPR, social support, and motivational interviewing frameworks are applied to test treatment effects of a two-phased CBPR walking intervention, including a 6-month active intervention quasi experimental phase and 12-month maintenance randomized controlled trial phase to test dose effects of motivational interviewing. A community advisory board helped develop and execute the culturally-appropriate intervention components which included social support walking groups led by peer coaches, pedometer diary self-monitoring, monthly diet and physical activity education sessions, and individualized motivational interviewing sessions. Although the study is on-going, three month data is available and reported. Analyses include descriptive statistics and paired t tests.

**Results:**

Of 269 enrolled participants, most were African American (94%) females (85%) with a mean age of 43.8 (SD = 12.1) years. Across the 3 months, 90% of all possible pedometer diaries were submitted. Attendance at the monthly education sessions was approximately 33%. At the 3-month follow-up 227 (84%) participants were retained. From baseline to 3-months, systolic BP [126.0 (SD = 19.1) to 120.3 (SD = 17.9) mmHg; p < 0.001] and diastolic BP [83. 2 (SD = 12.3) to 80.2 (SD = 11.6) mmHg; p < 0.001] were significantly reduced.

**Conclusions:**

This CBPR study highlights implementation factors and signifies the community's active participation in the development and execution of this study. Reach and representativeness of enrolled participants are discussed. Adherence to pedometer diary self-monitoring was better than education session participation. Significant decreases in the primary blood pressure outcomes demonstrate early effectiveness. Importantly, future analyses will evaluate long-term effectiveness of this CBPR behavioral intervention on health outcomes, and help inform the translational capabilities of CBPR efforts.

## Background

Community-based participatory research (CBPR) has been recognized as an important approach to develop and execute health interventions among marginalized populations, and a key strategy to translate research into practice to help reduce health disparities[1]. The CBPR approach is designed to ensure community participation in all aspects of the research process and build equitable community-academic partnerships[2]. Gaining trust in vulnerable communities and promoting intervention program sustainability have been noted as essential elements of CBPR. Despite growing interest in the CBPR approach, the literature reveals numerous deficits related to evaluating health outcomes[2,3]. Most notably, CBPR research initiatives rarely use experimental or other rigorous research designs[4,5]. For example, in a seminal systematic review, only 12 of 60 CBPR studies evaluated an intervention, and of these only four were randomized-controlled trials[2]. This report concluded insufficient evidence and too much variation to establish effectiveness among reviewed CBPR studies[2]. In a more recent critical review of both empirical and nonempirical participatory research literature, a similar conclusion was noted including lack of consistency in the use, measurement, and reporting of both process and outcome measures[3]. To accurately evaluate the translational capability of CBPR efforts, experimental approaches that consider and report on validity issues and establish both short- and long-term effectiveness of intervention efforts on health outcomes are needed[6,7].

CBPR creates an interesting paradox between balancing internal and external validity issues that can further obscure long-standing and complicated questions surrounding validity issues within effectiveness trials[3,7-10]. Importantly, CBPR is founded upon data sharing and community dissemination efforts, as well as utilizing culturally appropriate recruitment strategies, intervention approaches, and measurement instruments. However, these founding principles can simultaneously compromise internal validity issues, as well as threaten external validity and the generalizability of research outcomes to other communities. For scientific advancement of the CBPR field, there is a need for increased attention to and reporting of both internal and external validity factors, including but not limited to study design, measurement and instrumentation, statistical power, reach, effectiveness, adoption, implementation and maintenance[7,8,11]. Lack of scientific rigor in study designs and lack of attention to balancing internal and external validity issues may threaten dissemination of CBPR trials into the scientific literature, disqualify them from important systematic reviews, and consequently impede the funding and support of CBPR efforts.

The current study is a community-based participatory research (CBPR) walking intervention, H.U.B. City Steps, designed to address two of the most notable health priorities, a lack of physical activity and a high prevalence of cardiovascular disease (CVD), among African Americans residing in Hattiesburg, Mississippi. Unfortunately, racial/ethnic and state disparities are well-documented for this population for numerous CVD risk factors including hypertension (HTN), lack of physical activity, and overweight/obesity[12,13]. This study applies the CBPR[14], community capacity[15], social support[16,17], and motivational interviewing frameworks[18] to address three overarching research aims including: 1) to develop and assess community capacity to promote physical activity and healthy food choices, 2) to test treatment effects of a 6-month CBPR walking intervention on systolic blood pressure (SBP) among all walking participants, and 3) to test the dose effects of 4 versus 10 follow-up motivational interviewing telephone contacts on systolic blood pressure (SBP) over a 12-month maintenance phase. Although this study is on-going, the purpose of this paper is to describe the CBPR methodology of H.U.B City Steps, and provide early findings related to the reach, adoption, implementation, and effectiveness on primary blood pressure outcomes.

## Methods

### Study design and power calculation

All phases of this research were approved by The University of Southern Mississippi's Institutional Review Board, and informed consent and a medical disclaimer were obtained from all participants upon enrollment into the study. To provide benefit to all community participants and advance the scientific rigor of CBPR trials, this CBPR study utilized a 2-phase design approach. The first 6-month intervention phase was a quasi experimental design to assess the effectiveness of intervention treatment effects on SBP. During this phase, all enrolled walking participants were offered the same 6-month intervention which included social support provided by walking group volunteer leaders (designated "coaches"), motivational interviewing provided by intervention staff, pedometer diary self-monitoring, and five monthly education sessions. The second 12-month maintenance phase is a randomized controlled trial to assess the treatment effects of a low versus high dose of motivational interviewing delivered via telephone. Over the 12-month maintenance phase, the low treatment arm will receive four additional telephone motivational enhancement sessions while the high treatment arm will receive 10 additional motivational enhancement sessions. Between the two groups, the motivational enhancement sessions are designed to be of similar content, quality and session length.

This study was powered to test the treatment effects of the motivational interviewing dose at 18-months on SBP, while controlling for 6-month intervention treatment effects. Anticipating a 20% attrition rate at 18-months, 267 participants were enrolled. The projected sample at 18-months (N1 = N2 = 106) provides 80% power to detect a moderate effect size of 0.4 [difference of 6 (SD = 15) mmHg between groups] with an alpha of 0.05.

### Hattiesburg community & targeted population

Hattiesburg, located in southeast Mississippi, has a population of approximately 45,000 residents. The median household income of Hattiesburg is $24,409, which is lower than state and national averages at $31,330 and $41,994, respectively [19]. The city is approximately 47% African-American and 49%White. Twenty-four percent of deaths among non-whites in 2007 in Hattiesburg were attributable to heart disease, cerebrovascular disease, and stroke, but no incidence or prevalence data are available for these or other conditions at the city or county level [13]. For non-whites in the nine county southeast Mississippi public health district in which Hattiesburg is located, the prevalence rate for hypertension in 2007 was 43%[12].

Recruitment efforts were primarily directed toward African American residents; however race/ethnicity was not an exclusion factor. Eligibility criteria included 18 years of age or older, English-speaking, non-institutionalized, and residing in the Hattiesburg area. Participants with blood pressure over 180/110 were directed to obtain immediate medical attention and were disqualified from participating; however, all other participants were eligible for study participation regardless of blood pressure status and medication regimen.

### Engagement of community members and the community-based participatory process

HUB City Steps was planned in the context of a community-wide wellness initiative, Get Healthy Hattiesburg (GHH), led by the mayor's office. One of the priorities of the GHH steering committee was to increase physical activity in the community. The city of Hattiesburg and its GHH steering committee collaborated with the University of Southern Mississippi in preparing the grant application that funded HUB City Steps. University researchers shared with the GHH steering committee information about the design and outcomes of a feasibility study and pilot test of a community walking intervention successfully conducted in a rural Mississippi community [20-24]. Together, these collaborators adapted the 6-month quasi-experimental intervention design previously used by proposing in the grant application to target members of neighborhood associations in majority African American city wards, a recruitment approach that was already being used by GHH. When the HCS CBPR intervention project was funded, GHH steering committee members were invited to become part of a community advisory board (CAB) for HCS. In configuring the HCS CAB, HCS project staff also recruited as members other individuals who were engaged in community networking and advocacy/service provision within the targeted African American community in Hattiesburg, and/or in health promotion in the broader community. Invitations were issued to an initial organizational meeting that took place in November 2008, at which attendees were oriented to the concepts of CBPR and to the pilot intervention. Membership stabilized at 21 members, who are affiliated with local city and county government (n = 5), public and private health and medical clinics and agencies (n = 9), organizations with an educational mission, including the local school district and the Extension Service (n = 4), and private non-profit community organizations (n = 3). Twenty members are female, 14 African American (AA) and 7 White, and the median age range of members is 31-40.

During its first seven months of monthly meetings, the CAB created a community capacity building action plan for physical activity, and as part of that plan, completed such tasks as creating a vision and mission statement, naming the intervention, and designing a logo. The community capacity building action plan, described elsewhere[15], utilized Lempa and colleagues' framework for community capacity[16] and applied lessons learned related to community capacity through the prior pilot intervention[25]. The CAB also took on the responsibilities of providing oversight and guidance related to intervention implementation, evaluation, and planning for sustainability. As part of the action plan, the CAB identified community gatekeepers related to the HUB City Steps intervention. These were individuals who were perceived by the CAB as having the potential to influence the development or success of the intervention, by virtue of their holding positions of respect or influence within health, government, education, neighborhood, social service and church settings in the African American community or the larger geographic community of Hattiesburg. For the purpose of intervention adaptation and tailoring, community gatekeepers were invited to attend one of three community conversation workshops held during July-August 2009, and a similar workshop was held with members of the Community Advisory Board. The workshops were facilitated by a research staff scientist with extensive experience in community health planning, and used a focus group type methodology (unpublished data). The community conversations elicited input on a variety of aspects of intervention planning, including recruitment of walking coaches and participants, retention of participants, intervention design, scheduling and format of education sessions, and data collection procedures. Participants in the four community conversation sessions numbered 25, of which 20 were African American and 23 were female. Results summaries were provided to the research staff and recommendations were incorporated into protocols and manuals of procedures. For example, the maintenance phase design was modified to randomize participants to a low or high dose motivational interviewing-based telephone follow-up, rather than randomizing half the participants to a reminder-only maintenance group to receive quarterly calls about health fair opportunities.

### Conceptual framework

In addition to CBPR and community capacity, social support and motivational interviewing frameworks were used to undergird this project. These frameworks share something of a common orientation in that each focuses away from the trained professional and towards the individual or community owning the problem or challenge. For examples, social support emphasizes the role of family, friends, and community to provide comfort and encouragement[18,26], and MI calls forth the individual's problem-solving skills to address behavior change[19]. Research indicates that social support predicts increased physical activity either directly or indirectly through its impact on self-regulation and self-efficacy[27-31]. Including social support as a component in community physical activity interventions has been strongly recommended by the Task Force on Community Preventive Services based on a systematic review of physical activity interventions[32,33]. Social support has also been shown to be an indirect predictor of maintaining long-term physical activity and has been found to be an important psychosocial construct in behavioral intervention research in African American populations[34-36].

Motivational interviewing (MI) is a collaborative, participant centered counseling approach aimed at strengthening internal motivation to change a behavior[19,37]. Developed as a non-confrontational style, MI represents an important shift in addressing behavioral change by focusing on evoking an individual's reasons, needs, and desires for change while recognizing that ambivalence about change is natural and must be resolved by the individual[38]. MI has been studied extensively and shows promise as an efficacious intervention in diverse settings and with an assortment of health issues[39]. Although these studies generally support the use of MI as an approach for improving dietary and physical activity behaviors[40], there are some limitations to the existing body of research. For instance, more examination of MI's efficacy relating to health behavior promotion among low income, minority, and rural participants is warranted[41]. Furthermore, little is known about what MI dose is needed to promote and sustain health outcomes, and there are no known empirical studies that have examined the effectiveness of different MI doses[42].

### Researchers, intervention staff, and delivery agents

The research staff included contributions from eight doctoral level researchers (KY, JZ, CC, BW, MM, EM, CL, MW) and three master's level researchers (DC, AS, KZ). The community intervention staff included three full-time master's level African American women including an intervention coordinator who served as the primary liaison between the CAB and academic team, and also supervised the coach recruitment and retention coordinator and participant recruitment and retention coordinator.

Outcome data collections were staffed by a variety of individuals from both community and academic settings. Seven AA and one White woman were employed as community research assistants, serving as greeters, check-in personnel and escorts during data collection sessions, and providing child care as needed. They underwent a two-hour training session which included content on intervention goals, methods, and schedule, as well as participant confidentiality.

Anthropometric, biological, and fitness variables were assessed by trained exercise science and kinesiotherapy students (n = 18; 9 male; 2 AA, 15 White, 1 Hispanic). All students had completed a credit course in Exercise Testing and Prescription which included skill development, practice and evaluation of competency by the course instructor for all anthropometric measures, blood pressure, and the 6-minute walk test. All data collectors were specifically trained on study assessment protocols and required to demonstrate proficiency to the data collection coordinator for this area. Total training time was approximately three hours. Four of the 18 students were also trained to assess blood parameters using the Cholestech analyzer. In a 60-minute session, they were instructed on use of the Cholestech and in sterile procedures in the collection of capillary blood and handling of hazardous waste. Each student was required to perform 2-3 analyses on other individuals before being certified to perform this assessment during study data collection.

Six psychology graduate students (5 female; 1 AA, 5 White) served as interviewers, along with project research staff. All interviewers received six hours of classroom training and interviewing practice with other interviewers as well as engaging in home study. Training content included general interviewing skills as well as instrument-specific training, with a question by question review and use of response hand cards, on all psycho-social and demographic questionnaires and the food frequency questionnaire. Each interviewer was observed by the trainer while completing a minimum of three practice interviews. A booster session was held prior to each subsequent data collection.

Five doctoral level psychology graduate students and three master's level registered dietitians provided the MI. Seven were female, two AA and six White, with an average age of 28.1 (SD = 4.7). The counselors had 2.1 (SD = 1.9) years experience providing general clinical services. Training of MI counselors followed suggestions offered by Madson, Loignon and Lane[43] and consisted of 16 hours of didactic and experiential activities aimed at building skill in MI. Finally, the MI counselors conducted three audiotaped mock sessions following the study protocols, which were reviewed by fellow MI counselors and by the MI Intervention Director (a member of the Motivational Interviewing Network of Trainers) to assess counselor fidelity to the protocols and to provide formative feedback. Throughout the intervention fidelity was monitored through multiple means. First, participants completed the Client Evaluation of MI which assesses the client perception of a counselor's MI behavior[44]. Next, MI counselors completed a session specific checklist of activities they were to implement in each meeting with a participant. Finally, MI counselors completed the Therapist Evaluation of Motivational Interviewing which aims to assess the counselors' perception of MI consistent behavior during each counseling session. Feedback was also provided throughout the intervention about fidelity to the protocol based on review of these measures.

Education sessions were led by four community health professionals (all female, two AA, two White) trained to conduct the nutrition education component of each session, assisted by two fitness instructors (both AA) who led the exercise component of each education session. The nutrition education session leaders, who were professional health educators or registered dietitians, participated in five hours of training conducted by research staff which focused on session content and its conceptual underpinnings, session format, and the rationale and procedures for maintaining fidelity to the session content. Each session leader practiced conducting an education session while adhering to the fidelity monitoring guide for the session and being observed and critiqued by her peer session leaders and training staff. Because each education session was delivered three times, research staff fidelity monitors provided formative feedback after the first of the three sessions.

### Coach recruitment & training

The community intervention staff recruited walking group leaders using flyers and word-of-mouth, assisted by the CAB, City of Hattiesburg collaborators, and community agency contacts. Beginning August 1, 2009, an online application was posted through the university Human Resources website to allow all individuals interested in becoming walking coaches an opportunity to apply for the position. Initially, 28 coaches were hired. Of those, 26 completed the orientation session. At the end of the intervention participant enrollment, 21 of the originally hired coaches remained and three replacement coaches were added, yielding 24 walking coaches (23 female; 23 AA, 1 White) when the intervention began. Walking coaches participated in 16 hours of training, which included content on program goals, intervention design, protocols and procedures, walking coach responsibilities and compensation, group leadership and motivation, and CPR and first aid.

### Participant recruitment

HUB City Steps was publicized broadly to the target community over the six months preceding the intervention kick-off. Community buy-in and awareness was aided through efforts of community intervention staff, city of Hattiesburg partners, CAB members, walking coaches, and other community stakeholders. Information about HCS was shared with community members in a number of ways, which included word of mouth and distribution of brochures and flyers by personal contact and through participation of HCS staff in other scheduled community events such as Night Out Against Crime and community health fairs. A HCS flyer provided to all potential participants during community events and recruitment activities conducted by staff and coaches used a Frequently Asked Questions format, describing each component and expectations for participation. The two phase approach was described, including the 6 month intervention phase and the assignment to one of two follow-up approaches during the 12 month maintenance phase.

As part of the walking coach training, each coach received training on participant eligibility and recruitment strategies, and was directed to recruit 10-12 persons to serve as walking participants on his/her team. Research staff and CAB members helped to identify potential participants. During the recruitment process, research staff compiled a membership list for each walking group, consisting of potential participants who had been nominated by a coach, staff member, or stakeholder, or who had self-identified as interested in participating. Final eligibility was determined using a pre-enrollment screening form, which was collected from each potential participant by her/his coach, and reviewed by project staff to determine eligibility and need to obtain a medical release to participate, required of anyone who answered yes to one or more of five screening questions.

### Intervention components

#### Coaches group leadership and social support

During the recruitment phase, walking coaches identified and contacted potential group members, informed them, as part of the informed consent process, of the responsibilities of joining HUB City Steps, provided and collected pre-enrollment screening forms, and advised and reminded group members regarding making enrollment appointments. Once participants were enrolled, coaches performed the following functions for their walking groups: contacted each group member weekly to encourage routine walking; worked with each participant to set individual weekly walking goals; encouraged each group member to walk throughout each week; arranged group walking and health-related activities at their discretion (minimum of two per month required); monitored each group member in completing and submitting walking records; notified group members about upcoming educational sessions and data collection health assessments; served as a liaison between walking group members and project staff by conveying any questions or problems to the staff. The coaches were also required to perform all the responsibilities expected of participants, including submitting walking logs and attending data collection health assessments and education sessions.

Coaches were paid at a rate of $15.00 per hour for training, recruitment of group members up to a total of 15 hours, and for walking group leadership activities up to 8 hours per week during the intervention phase. Coaches maintained participant contact logs, and submitted biweekly time sheets to the community coordinator. Coaches were provided with biweekly progress reports on their group members' activity which were generated from the submitted walking logs.

#### Pedometer diary self-monitoring

Each participant received a Yamax pedometer (Yamax model SW-701, Yamax corporation, Tokyo, Japan), and the intervention staff provided face to face instruction on pedometer use and individualized stride calibration. Participants were instructed to wear the pedometer on their waist during waking hours, to remove only upon showering, bathing or swimming, and to reset the pedometer to zero each morning. Participants had the option of recording their daily steps on weekly pedometer diary postcards or recording daily steps through logging on to the intervention's website. Coaches were responsible for encouraging their team members to record their step counts and submit their weekly diaries. Monthly step count reports that illustrated each team members' step count, as well as the total team's step count, was generated and distributed to each group at the monthly education sessions.

#### Education sessions

The monthly education sessions lasted approximately 90 minutes and included a group physical activity, sharing time for successes and challenges, education focused on the principles of the DASH diet[45], and time for questions and evaluations. Each monthly session was offered three times at varying times, days of the week, and location to accommodate participants' schedules and encourage broader participation. The Transtheoretical Model of Change, and most notably the processes of change, were used to guide development, execution, and evaluation of the education sessions[46]. In brief, the monthly education sessions focused on: 1) the negative side effects of high blood pressure and the benefits of exercise in controlling BP; 2) the DASH diet and community options available for exercise and healthy eating; 3) problem solving techniques related to cooking and the role of sodium in controlling blood pressure; 4) empowering the participant to implement healthy lifestyle changes; and 5) healthy choices when eating out and identifying triggers that may influence unhealthy menu choices. A sixth monthly session was a celebration event. Attendance was recorded at each session and session evaluations were completed by attendees.

#### Motivational interviewing

The outcome measures described below were framed as a health assessment for the participants. Immediately upon completing the health assessment, a 'Know Your Numbers' card was provided to each participant which detailed individual blood pressure, anthropometric and biological results, and dietary intake. This card served as a central point of discussion for MI. Participants engaged in a one-on-one MI session aimed at building participant internal motivation to adopt health eating and exercise behaviors and to develop an individualized change plan at baseline, which was reviewed and updated at subsequent data collection time points.

### Outcome Measures

All outcome measures are scheduled at baseline, 3 months, 6 months and 18 months (or 12 months post-intervention). A data collection manual of procedure was developed to detail standardized guidelines for assessing all outcome measures. Data collection occurred at a local community center, conveniently located for study participants.

#### Medical history, background, and dietary intake

A medical history questionnaire was used to assess diagnosis and prescribed medications related to blood pressure, blood sugar and cholesterol. A medication adherence questionnaire was also added at the 3-month time point. Additional background information included fasting and smoking questions along with demographics. The National Cancer Institute's (NCI) Five-Factor Screener was used to monitor dietary intake. This valid 18-item screener approximates intake of fruits and vegetables servings, grams of fiber, teaspoons of added sugar, milligrams of calcium, and dairy servings[47,48].

#### Anthropometric, biological, and fitness variables

Blood pressure, height, weight, body composition, lipids, glucose, and the 6-minute walk test were assessed. Systolic and diastolic blood pressure was assessed using an OMRON HEM-907XL automatic inflation sphygmomanometer. Height was measured using a portable stadiometer and a Tanita Body fat analyzer model TBF-310T was used to measure weight and percent body fat and to calculate body mass index (BMI). Non-fasting cholesterol, triglycerides and glucose were assessed using the Cholestech LDX Lipid Analyzer. As a measure of fitness, the 6-minute walk test was completed. The 6-minute walk test (6MWT) is an objective, simple, inexpensive, and relatively safe exercise test and has been identified as the test of choice when using a functional walk test for research purposes[49,50]. The self-paced 6MWT assesses the submaximal level of functional capacity by having participants cover as much distance as possible on a hard, flat, 120 meter circuit within six minutes without running. The 6MWT has been shown to have high reliability and high ability to discriminate between functional levels in a high-function population[51].

#### Psychosocial measures

Previously validated psychosocial instruments were used and adapted for this study and included social support for physical activity[52], treatment self-regulation for physical activity and treatment self-regulation for diet[53], and processes of change for physical activity[54]. The adapted instruments included physical activity social support from coaches (11 items, 3-sub-scales: guidance, reliable alliance, reassurance of worth); physical activity social support from walking group members (12 items, 3-sub-scales: guidance, reliable alliance, social integration); treatment self-regulation for diet and physical activity (15 items each, 4-sub-scales each: amotivation, external, introjection, identification and integration); and processes of changes (30 items, 10 subscales: stimulus control, social liberation, reinforcement management, helping relationships, counter conditioning, self-liberation, self-reevaluation, environmental reevaluation, dramatic relief, and consciousness raising). Two phases of formative testing were conducted with individual's representative of the target population on the adapted psychosocial instruments, including cognitive testing with five individuals and pilot testing with 20 individuals. Feedback resulted in wording, question structure, and format changes and promoted cultural appropriateness of survey items for use with the target population. Cronbach's alphas and item analysis statistics were used to evaluate the internal consistency of the psychosocial instruments on the baseline data. The majority (24 of 29) of scales and subscales demonstrated strong internal consistency with Cronhbach's alpha ≥0.70 when item composition was maintained from the intended item clustering. Moderate internal consistency ranging with Cronhbach's alpha from 0.44-0.63 was noted for five scales including amotivation for physical activity, amotivation for diet, dramatic relief, self-liberation, and social liberation.

### Data analyses

Descriptive statistics including means, standard deviations, frequencies, and percents were used to summarize demographics, participation and compliance rates, and outcome variables. Paired t tests were used to examine the difference in blood pressure between baseline and 3-month follow-up among all study participants. A further subgroup analysis was conducted to examine the difference in blood pressure among participants with normal blood pressure, participants diagnosed with high blood pressure and taking medication, and participants diagnosed with high blood pressure but not taking any medication in the past 30 days. All statistical analyses were performed using the SAS 9.1.3 statistical software package (SAS Institute Inc., Cary, NC). A critical value of .05 was adopted for significance testing in bivariate comparisons.

## Results

### Study sample

Of the 269 enrolled participants, most were African American (94%) females (85%) with a mean age of 43.8 (SD = 12.1) years (Table 1). Additional demographic information including marital status, socioeconomic status, BMI, and self-reported health status are detailed in Table 1. Half of the participants had completed a college education and the approximated average annual income was $32,200. Of all participants, 90% were categorized in an unhealthy BMI status. In terms of representativeness, this study was specifically designed to target a higher proportion of African Americans. Recruitment efforts resulted in a higher proportion of women than men. The average annual income of enrolled participants was comparable to state averages ($31,330), but higher than regional averages ($24,409). The prevalence of hypertension among enrolled participants at baseline (42%, see Table 2) was nearly identical to the targeted region of 43%.

**Table 1:** Socio-Demographic Characteristics of Study Sample

	frequency	Percentage
N (%)	269	100%
Sex		
Male	40	14.9%
Female	229	85.1%
Age (mean ± SD)	269	43.8(12.1)
Race		
Black or African American	254	94.4%
White	14	5.2%
American India or Alaska native	1	0.4%
Education		
<11^th ^grade	12	4.5%
12 grade (high school grad/GED)	41	15.2%
Trade or VOC school	13	4.8%
Some college	61	22.7%
College degree	76	28.3%
Some graduate or professional school	19	7.1%
Graduate level or professional degree	47	17.5%
Total income in the last 12 months		
Less than $9,999	40	14.9%
$10,000-$19,999	36	13.4%
$20,000-$29,999	54	20.1%
$30,000-$39,999	37	13.8%
$40,000-$49,999	30	11.2%
≥$50,000	71	26.4%
Marital status		
Now married	113	42.0%
Widowed	12	4.5%
Divorced	47	17.5%
Separated	8	3.0%
Never married	89	33.1%
BMI		
Normal (18.5-24.9)	26	9.7%
Overweight (25-29.9)	52	19.3%
Obese (30-34.9)	77	28.6%
Morbidly obese (≥35)	114	42.4%
Health status		
Excellent	19	7.1%
Very Good	54	20.1%
Good	130	48.3%
Fair	60	22.3%
Poor	6	2.2%

Note: SD = standard deviation

**Table 2:** Changes in blood pressure between baseline and 3-month follow-up

Blood pressure	Baseline	Follow-up	t	p
All participants (n)	(n = 269)	(n = 227)		
Systolic blood pressure (mean ± SD)	126.0 (19.1)	120.3 (17.9)	6.01	<0.0001
Diastolic blood pressure (mean ± SD)	83.2 (12.3)	80.2 (11.6)	3.79	0.0002
*Stratified by blood pressure diagnosis and medication history*				
Participants with normal blood pressure (n)	157	120		
Systolic blood pressure (mean ± SD)	121.4 (17.9)	114.9 (16.3)	3.93	0.0001
Diastolic blood pressure (mean ± SD)	81.2 (12.0)	77.6 (10.9)	2.79	0.0061
Participants diagnosed with high blood pressure and taking medication (n)	94	88		
Systolic blood pressure (mean ± SD)	131.6 (18.8)	125.6 (17.8)	4.83	<0.0001
Diastolic blood pressure (mean ± SD)	85.7 (12.3)	82.7 (12.3)	2.70	0.0082
Participants diagnosed with high blood pressure but not taking medication (n)	18	19		
Systolic blood pressure (mean ± SD)	136.4 (20.1)	129.4 (17.7)	0.39	0.6997
Diastolic blood pressure (mean ± SD)	87.7 (11.0)	85.6 (7.8)	0.24	0.8123

### Three-month participation and retention rates

Across the first 3 months, 90% of all possible pedometer diaries were submitted. Compliance rates for maintaining and submitting pedometer diaries reveal that 33.8% of participants turned in 100% of their weekly pedometer diaries and 54.6% were at least 80% compliant with submitting weekly pedometer diaries. Attendance at months 1, 2, and 3 education sessions were 33.1%, 34.9%, and 32.7%, respectively. Among participants, 34.8% attended one session, 29.6% two sessions, and 35.6% three sessions. At the 3-month follow-up data collection 227 (84%) participants were retained. Although there were no significant differences in sex distribution, race, education, marital status, income and health status between the participants who enrolled and those retained at 3-months, young participants were significantly more likely to drop out. All 269 participants at baseline and 227 participants at the 3-month follow-up participated in the individualized MI sessions.

### Three-month preliminary BP outcomes

From baseline to 3-months, systolic and diastolic BP were significantly reduced by about 6 and 3 mmHg, respectively (p < 0.001) (Table 2). In subgroup analyses participants with normal blood pressure and participants diagnosed with high blood pressure and taking medication in the past 30 days achieved a similar significant reduction in both systolic and diastolic blood pressures. However, no significant blood pressure differences were found when the analyses were restricted to participants diagnosed with high blood pressure but not taking any medication in the past 30 days.

## Discussion

This study details important conceptual and methodological approaches of a CBPR intervention to reduce BP in an African American community, and establishes early findings related to the implementation, reach, adoption, and effectiveness. This study highlights numerous implementation factors and signifies the community's broad and active participation in the development and execution of this study, as well as important information related to the settings and delivery agents. In terms of reach and representativeness, utilizing community coaches to recruit participants resulted in an adequate sample size. The fact that women and higher SES residents were more likely to participate is a similar phenomenon noted across health behavior research studies, yet is important to consider in the generalization of findings to other communities. Future efforts are needed to understand factors that impact engagement as well as barriers to participating in CBPR interventions among men and lower SES residents. Preliminary adoption data indicate that adherence to pedometer diary self-monitoring was better than participation in the education sessions. Early effectiveness is demonstrated through the significant decrease in the primary blood pressure outcomes. Future analyses will include evaluation of 6- and 18-month (maintenance) outcome variables including the described anthropometric, biological, and fitness, psychosocial, and dietary variables. Process data including fidelity to treatment or up-take of intervention components will also be evaluated to determine effectiveness on targeted health outcome.

Among all participants in our study the statistically significant 3-month effect size was about 0.30 for systolic blood pressure and 0.25 for diastolic blood pressure. The effect sizes of the sub-group analyses were remarkably similar, yet the group of participants diagnosed with high blood pressure but not taking medication did not achieve a statistically significant change. As a noted study limitation, this sub-group contained a very small sample size and our study was not specifically powered for sub-group analyses. Premier is one of the most widely published clinical trials targeting blood pressure via diet and lifestyle changes[55-57]. A number of other randomized studies have demonstrated the effectiveness of increasing physical activity in the form of walking on lowering CVD risk factors such as systolic and/or diastolic blood pressure[58-61], although not all have[62]. Less is known about community-based participatory research efforts to improve CVD risk factors. Pazoki and colleagues utilized CBPR to design and implement an 8-week physical activity intervention among 335 Iranian women randomized to control and intervention groups and showed a decrease in systolic blood pressure of 10.0 mmHg within the intervention group[63]. However, Wilcox and colleagues were unable to demonstrate any effect on outcome measures utilized in a group randomized CBPR trial of physical activity among church members in African American churches in South Carolina that lasted 1-2 years[64]. Farag and colleagues utilized a quasi-experimental design along with CBPR to design and implement a 6-month physical activity intervention among school employees in Oklahoma and showed a 5.0 mmHg reduction among participants[65]. Our experimental CBPR trial to explore the short- and long-term effectiveness of a community-based trial is unique in its approach of incorporating motivational interviewing. The magnitude of change seen at the 3-month measure is clinically meaningful in reducing cardiovascular disease (CVD), especially in a population that is primarily African American[66,67], and compares to findings from the NIH-sponsored Dietary Approaches to Stop Hypertension (DASH) and the PREMIER trials[56,57].

While there are numerous challenges when collaboratively developing CBPR studies, one of the most notable is to employ a rigorous study design, yet ensure the direct research benefits are maximized for all participants and the larger community. The majority of CBPR studies tend to be non-experimental or single group quasi experimental designs[3], because a traditional multi-group randomized controlled trial may pose serious threats to the successful recruitment of participants, maximizing benefits to all participants, and cross-contamination among study groups[10,11]. While some have argued that RCT is not appropriate for CBPR trials[5], others have called for increased rigor in the design of studies[6]. Adequately powering a CBPR study can also be difficult. Contrary to the rigid inclusion/exclusion criteria of typical controlled clinical trials [i.e. blood pressure >120/80, antihypertensive medication criteria][55], community-based interventions such as this one, where the focus is on improving the health of the larger community, set inclusion/exclusion criteria to allow for involvement of nearly all interested community members. Consequently, increased variability and large standard deviations increases the sample size needed to determine statistically significant changes on targeted health outcomes. In this community where 90% of enrolled participants were at an unhealthy weight, the community clearly recognizes that hypertensive community members are not the only people who may benefit from intervention efforts to increase physical activity and improve dietary quality. Our two- phased research design including a 6-month quasi experimental phase followed by a 12-month RCT allows all community members to equally benefit from the structured intervention components, allows investigation of a multi-component structured CBPR intervention across numerous health outcomes, and also allows exploration of unanswered questions regarding the dose effects of MI as a vehicle for fostering participant adherence to healthy behavior change efforts after completion of a structured intervention.

Finally, although not described in detail in this paper, the evaluation of this walking intervention is contextualized under a larger evaluative framework that includes the development of community capacity. As one example, this research heavily focused on the development of practical and research skills of the community including engaging and empowering community members to participate in multiple levels of this research project, as well as providing experiential opportunities to academic faculty and students. This cogenerative learning is a fundamental principle of action research[68]. Similarly, promoting co-learning and capacity-building among partners is a fundamental principle of CBPR, as are shared decision-making power and mutual ownership of the research process and products[3,69]. Although community capacity building has been described as an essential element for reducing health disparities and can serve as an indicator of health intervention program sustainability [70-72], empirical data regarding the effectiveness of community coalitions is largely inconclusive[73]. Several key public health questions remain unanswered, for examples what is the value added benefits of utilizing a CBPR approach, what is the cost-effectiveness of CBPR, and what evidence suggests that CBPR leads to improved and maintained health outcomes and sustainability of partnerships. As the science of CBPR advances and demand for funding opportunities increases, researchers much be positioned to address these important, yet difficult, questions to address the translational capabilities of CBPR. Utilizing community-academic teams to operationalize independent and dependent variable associated with community capacity and coalition effectiveness at the onset of the partnership and intervention development is critical.

## Conclusion

In conclusion, CBPR recognizes that research is more than just answering questions about the statistical significance of the study's health outcomes. The social changes, increased community capacity, and increased accessibility to health programs are also important processes and outcomes. However, to advance the CBPR field, there is a need for more experimental research designs examining the effectiveness on health outcomes, with focused attention to and reporting of both internal and validity factors. Although this study is limited by its 3-month outcome data, it provides important practice implications for other CBPR initiatives for planning and reporting on study design, measurement and instrumentation, statistical power, reach, effectiveness, implementation, and adoption. The intersection of conventional translational science, traditional social science, and action research will continue to fuel the debate of internal and external validity. Negotiating middle ground to balance internal and external validity in CBPR is a notable, yet worthy challenge, especially among health disparate communities where community voices are frequently underrepresented.

## Competing interests

The authors declare that they have no competing interests.

## Authors' contributions

JZ and KY conceptualized and drafted the paper. Each author contributed to further development and revisions of the paper, and approved the final submission. KY, JZ, CC, and MM contributed to securing grant funding for the project and conceptualization of the study design, measurement, and evaluation. Each author assumed a unique role in execution of the intervention including: JZ-data and evaluation manager, CC-intervention manager, MM-motivational interviewing coordinator, BW-statistician, VR-recruitment and retention manager and community advisory board liaison, EM-education coordinator, and KY-principal investigator.
